# mRNA Innovates the Vaccine Field

**DOI:** 10.3390/vaccines9050486

**Published:** 2021-05-11

**Authors:** Norbert Pardi

**Affiliations:** Department of Medicine, University of Pennsylvania, Philadelphia, PA 19104, USA; pnorbert@pennmedicine.upenn.edu

Development of new vaccine modalities is a critical need to address many of the shortcomings of traditional platforms such as the lack of sufficient efficacy against certain pathogens or cancer; difficulties with production; or, in some cases, safety issues. The concept of mRNA vaccines has been around for decades but many obstacles that hindered the generation of safe and effective mRNA vaccines have only recently been overcome [1]. The SARS-CoV-2 pandemic created an unexpected opportunity for the technology to demonstrate its promise. Indeed, mRNA-based vaccines against SARS-CoV-2 were generated and evaluated very quickly (11 months after the start of development) and received approval for human use in December 2020. As of April 2021, more than 100 million doses of SARS-CoV-2 mRNA vaccines have been administered worldwide, contributing to decreasing morbidity and mortality caused by COVID-19. As the guest editor of the Special Issue of Vaccines entitled “The past, present, and future of mRNA vaccines”, I feel honored to receive publications from respected leaders of the field of mRNA vaccines. This valuable collection covers the latest findings of infectious disease and cancer mRNA vaccines, discusses the mechanisms of immune activation of mRNA vaccines, and also highlights the challenges regarding regulatory practices and manufacturing.

The SARS-CoV-2 pandemic has caused a worldwide disaster, killing more than three million people as of April 2021 and causing significant economic problems. Vaccine development against the virus was extremely fast, and several approved vaccines for humans have become available in the past several months. SARS-CoV-2 mRNA vaccines are playing a key role in battling the pandemic. In this issue, Bettini and Locci reviewed the latest findings on SARS-CoV-2 mRNA vaccines, discussed strategies for antigen design and recent preclinical and clinical data, and provided an overview about immunological mechanisms induced by this vaccine type [2].

Flaviviruses such as Zika, Dengue, and West Nile viruses cause significant morbidity and mortality throughout the world. After the serious Zika virus outbreak in 2015–2016, several proof-of-concept studies demonstrated that mRNA vaccines could induce protective immune responses against flaviviruses [3]. In a detailed review, Wollner and Richner summarized the published data on this topic [3].

HIV infection is a global threat and, despite the investment of enormous research efforts into HIV vaccine development in the past 40 years, no approved vaccine is available against the virus. HIV is certainly one of the most difficult vaccine targets as it has multiple mechanisms to escape from the antiviral immune responses. Mu et al. not only summarized the latest results obtained with HIV mRNA vaccines but provided a detailed overview on the challenges and potential strategies for developing effective vaccines against HIV [4].

mRNA vaccines represent a promising platform for the development of therapeutic cancer vaccines because they can induce potent T cell responses and they can be quickly modified, if necessary [5]. The flexibility of mRNA vaccine design is particularly important for personalized neoantigen cancer vaccine development since unique antigens need to be produced for each cancer patient. Esprit and de Mey et al. reviewed the history, general concepts, and current standing of the field of neoantigen mRNA cancer vaccines. Importantly, the article provides a detailed overview about clinical studies and companies that are using this novel, innovative approach.

The self-amplifying mRNA (saRNA) vaccine platform has proved to be potent in multiple preclinical studies, and the safety and efficacy of saRNA vaccines are currently being investigated in clinical trials. In a detailed review paper, Blakney et al. discussed new developments, key considerations, and challenges for the successful development of saRNA vaccines against cancer and infectious diseases [6].

Although multiple publications have demonstrated the outstanding efficacy of mRNA vaccines in preclinical models and people, our knowledge is limited on the mechanisms of action and characteristics of immune responses elicited by this vaccine modality. Cagigi and Loré reviewed published data on the mechanism of innate immune activation, antibodies, and T cell responses induced by mRNA vaccine administration [7]. Additionally, they comment on the strengths and potential weaknesses of mRNA vaccines compared to traditional vaccine platforms.

Monoclonal antibody therapy is gaining increasing attention because it can offer a highly specific and effective treatment for cancer, pathogenic infections, autoimmune diseases, and other medical conditions. The vast majority of published mRNA vaccine studies have utilized the platform for active immunization but several studies have provided proof-of-concept on the suitability of mRNA-based modalities for passive immunotherapy [8]. Deal et al. reviewed the latest advancements in mRNA-encoded antibody development and discuss antibody designs, the range of applicability, and the limitations of this novel therapeutic approach [8].

Safe and efficient in vivo delivery of mRNA is a critical consideration for therapeutic applications. Buschmann et al. provided a detailed overview about delivery materials for mRNA vaccines [9]. The publication focuses on lipid nanoparticles (LNPs), as they are currently the most widely used type of mRNA carrier. The authors describe the history of LNPs, review SARS-CoV-2 vaccine trials that use LNP-encapsulated mRNA vaccines, and discuss key considerations (dose, route of delivery, endosomal release etc.) that influence the efficacy of mRNA–LNP vaccines.

Production of a sufficient number of vaccines in a short time is an extraordinary challenge during a pandemic as experienced at present. SARS-CoV-2 mRNA vaccines are used in multiple countries worldwide and, therefore, play a key role in ending the COVID-19 pandemic. In an interesting study, Kis et al. assessed the production process, cost, scale, and time needed for manufacturing mRNA vaccines under a pandemic scenario [10]. They examined these factors for three nonreplicating mRNA vaccines (Moderna, Pfizer-BioNTech, and CureVac) and one saRNA (Imperial College London) vaccine.

In order for vaccines to get to market, regulatory agencies need to evaluate the preclinical and clinical data and approve them as safe and effective for human use. Knezevic et al. briefly reviewed mRNA vaccine technologies and summarized important considerations regarding the evaluation of mRNA vaccines by regulatory perspectives [11]. The authors discussed the regulatory requirements for mRNA vaccines that should be established by national regulatory agencies on the basis of WHO guidelines or recommendations.

Taken together, this special issue contains 10 timely and highly relevant research and review articles focused on various aspects of mRNA vaccines. These contributions and the recent success of the SARS-CoV-2 mRNA vaccines suggest that this novel vaccine modality holds immense promise, potentially revolutionizing the field of infectious disease and cancer vaccines.

## Data Availability

Not applicable.
